# The effects of cytisinicline for smoking cessation on arterial stiffness, endothelial function, and myocardial performance: a pilot study

**DOI:** 10.1093/ehjimp/qyaf062

**Published:** 2025-05-19

**Authors:** Ignatios Ikonomidis, John Thymis, Gavriella Kostelli, Konstantinos Katogiannis, Dimitrios Vlastos, Eleni Gatourtzidou, Kallirhoe Kourea

**Affiliations:** 2nd Cardiology Department, University Hosptal Attikon, National & Kapodistrian University of Athens, Rimini 1, Haidari, Athens 12462, Greece; 2nd Cardiology Department, University Hosptal Attikon, National & Kapodistrian University of Athens, Rimini 1, Haidari, Athens 12462, Greece; 2nd Cardiology Department, University Hosptal Attikon, National & Kapodistrian University of Athens, Rimini 1, Haidari, Athens 12462, Greece; 2nd Cardiology Department, University Hosptal Attikon, National & Kapodistrian University of Athens, Rimini 1, Haidari, Athens 12462, Greece; 2nd Cardiology Department, University Hosptal Attikon, National & Kapodistrian University of Athens, Rimini 1, Haidari, Athens 12462, Greece; 2nd Cardiology Department, University Hosptal Attikon, National & Kapodistrian University of Athens, Rimini 1, Haidari, Athens 12462, Greece; 2nd Cardiology Department, University Hosptal Attikon, National & Kapodistrian University of Athens, Rimini 1, Haidari, Athens 12462, Greece

## Introduction

Cytisinicline is a plant-derived alkaloid that is a partial agonist of nicotinic acetylcholine receptors with an affinity for the α4β2 receptor.^1^ At the standard dose is considered an efficacious and safe treatment for smoking cessation.^1^ Cytisinicline has been shown to reduce oxidative stress and improve contractility of papillary muscles in hearts of animal models.^2^ Nonetheless, its effects on arterial stiffness, endothelial function, and cardiac performance have not been elucidated yet.

## Methods

In an observational case–control study, we recruited 60 consecutive smokers who attended our smoking cessation unit, of which: (i) 30 active smokers were commenced on cytisinicline at standard dosing regimen^1^ (WinMedica,A.E., Agisilaou, Marousi, Greece) and (ii) 30 active smokers who sought for consultation but were not eager to stop smoking and served as control group. We assessed both at baseline and at 1-month follow-up the smoking status, Fagestrom score (marker of nicotine addiction), arterial stiffness, endothelial function, and cardiac deformation. Smoking status was addressed by self-reported smoking burden per day and by measuring primarily exhaled carbon monoxide (eCO) concentration measurement [parts per million (ppm), Bedfont Scientific, Maidstone, Kent, UK]. The controls were matched with the cytisinicline group for age, sex, and pack-years. Exclusion criteria included history of cardiovascular or other systemic disease, use of any medication, and smoking less than five cigarettes per day in conjunction with eCO < 10 ppm (*Table 1*).

**Table 1:** Data are presented as mean ± standard deviation

	Group	Baseline	1 month	*P*-value
eCO (ppm)	Cytisinicline	16.51 ± 5.72	4.87 ± 2.01	<0.001
	Control	15.97 ± 6.11	16.23 ± 5.88	0.891
PWV (m/s)	Cytisinicline	10.36 ± 2.37	8.69 ± 1.94	0.019
	Control	9.87 ± 1.96	10.02 ± 2.23	0.822
cSBP (mmHg)	Cytisinicline	116.71 ± 10.29	110.46 ± 9.12	0.048
	Control	118.15 ± 11.25	117.36 ± 10.70	0.821
PP (mmHg)	Cytisinicline	40.91 ± 9.37	34.66 ± 8.52	0.033
	Control	42.17 ± 8.29	41.05 ± 9.07	0.685
PBR20-25 (μm)	Cytisinicline	2.55 ± 0.24	2.39 ± 0.19	0.024
	Control	2.58 ± 0.26	2.76 ± 0.29	0.045
FMD (%)	Cytisinicline	7.51 ± 1.26	11.03 ± 2.27	<0.001
	Control	7.36 ± 1.69	8.01 ± 1.25	0.140
GLS (%)	Cytisinicline	−19.83 ± 1.37	−21.68 ± 1.71	0.024
	Control	−19.67 ± 1.43	−20.41 ± 0.97	0.314
PWV/GLS	Cytisinicline	−0.54 ± 0.10	−0.40 ± 0.09	0.010
	Control	−0.50 ± 0.11	−0.49 ± 0.08	0.456
E/E′	Cytisinicline	7.01 ± 2.19	5.50 ± 1.89	0.046
	Control	7.12 ± 2.01	7.32 ± 2.38	0.679

To assess arterial stiffness, we calculated carotid–femoral pulse wave velocity (PWV), central aortic pressures (central systolic and diastolic), and pulse pressure (PP) using a tonometry device (Complior Alam Medical, Vincennes, France).

Endothelial glycocalyx is a glycoprotein cell layer that protects from circulating cells to endothelial cells interaction and endothelial permeability to cells and macromolecules. To address endothelial glycocalyx thickness, we estimated the perfused boundary region (PBR) of the sublingual arterial microvessels with a diameter range 20–25 μm employing Sideview Dark Field imaging (Microscan, Glycocheck, Microvascular Health Solutions Inc., Salt Lake City, UT, USA). The PBR reflects the cell-poor layer that results from the separation between the red blood cell column and plasma on the luminal endothelial surface. An increased PBR value is interpreted as vulnerable glycocalyx layer with reduced thickness.^3^ We also measured flow mediated dilation (FMD, %) of the brachial artery utilizing ultrasound to assess endothelial function.

To assess cardiac function, we measured by Doppler echocardiography the E wave deceleration time and early diastolic velocity (E′), at septal and lateral mitral annulus sites. The E/E′ ratio was calculated to assess diastolic function. By employing dedicated software (EchoPac206 PC, GE Healthcare), global longitudinal strain (GLS) was estimated from the 17-LV-segment model. We also measured the PWV/GLS ratio to assess ventriculoarterial interaction.

## Results

Participants in the cytisinicline group were 50 ± 8 years old, 18/30 (60%) were males and smoked 28 ± 7 cigarettes/day, 28 ± 8 pack-years, and the Fagestrom score was 7.71 ± 1.49. Participants in the control group were 48 ± 7 years old, 16/30 (53.3%) were males and smoked 27 ± 9 cigarettes/day, 27 ± 8 pack-years, and the Fagestrom score was 7.58 ± 1.53. None of the participants was under any medication at inclusion. Also, the baseline measurements of arterial stiffness, endothelial function, and cardiac performance did not differ significantly between cytisinicline and control groups (*P* > 0.05).

At follow-up, 29/30 cytisinicline users ceased smoking. During treatment, 18 participants reported nausea and 1 high blood pressure. However, none of the participants discontinued cytisinicline. Also, 1 month after completion of cytisinicline treatment, 29/30 participants still abstained from smoking.

Their eCO levels decreased significantly (*P* < 0.001) post-treatment while remained unchanged in the control group (*P* > 0.05).

Compared with baseline, administration of cytisinicline resulted in decrease of PWV, PP, and cSBP (*P* < 0.05 for all comparisons). Heart rate remained unchanged throughout the study. No difference of the above markers was observed in controls (*P* > 0.05).

PBR20-25 was improved in cytisinicline arm (*P* = 0.024), while worsened in the controls (*P* = 0.045). FMD increased only in cytisinicline arm (*P* < 0.001) and remained unchanged in controls (*P* > 0.05).

Participants in cytisinicline group improved GLS, PWV/GLS, and E/E′ (*P* < 0.05). At follow-up, improved PWV/GLS was associated with improved E/e′ (*r* = −0.37, *P* = 0.031) in cytisinicline users. Also, improved PWV was associated with GLS (*r* = 0.30, *P* = 0.039). The above markers did not change in controls (*P* > 0.05).

The study design does not permit to assess causality between changes of the examined markers and treatment. Due to the small sample size, the current study should be considered as a pilot study. Larger-scale randomized controlled trials with longer follow-up periods are warranted to validate our findings.

## Conclusion

The use of cytisinicline in the standard regime for one month in smokers resulted in significant amelioration of endothelial function arterial stiffness, cardiac performance in conjunction with safe and effective smoking cessation compared with smokers who did not cease smoking for a similar time period. Thus, improved ventriculoarterial interaction may have led to improved cardiac function in cytisinicline users.

## Ethics

ClinicalTrials.gov Identifier: NCT06885970.

## Consent

Each participant provided a written informed consent before joining the study.

## Data Availability

The data underlying this article will be shared on reasonable request to the corresponding author.

## Lead author biography

Ignatios Ikonomidis, MD, PhD, is Professor of Cardiology, Director of Echocardiography, Laboratory of Preventive Cardiology and Clinic of Cardiometabolic Diseases, 2nd Cardiology Department, National and Kapodistrian University of Athens (NKUA), Attikon Hospital, Athens, Greece. In 1997, he completed his training in Cardiology and Cardiac Imaging, in Hammersmith Hospital, Imperial College, London, UK. Since then, he worked as consultant cardiologist in University Hospital of Patras, Department of Clinical Therapeutics and 2nd Cardiology Department, Medical School, Athens. His research fields include cardiovascular imaging, microcirculation, inflammation, diabetes, smoking control, and ventricular–arterial interaction. He has authored 330 publications in indexed journals and several book chapters.
